# Intima-Media Thickness Does Not Differ between Two Common Carotid Artery Segments in Children

**DOI:** 10.1371/journal.pone.0149057

**Published:** 2016-03-11

**Authors:** Heidi Weberruß, Raphael Pirzer, Robert Dalla Pozza, Heinrich Netz, Renate Oberhoffer

**Affiliations:** 1 Institute of Preventive Pediatrics, Technische Universität München, Munich, Germany; 2 Department of Pediatric Cardiology, Ludwig-Maximilians-University, Munich, Germany; Bambino Gesù Children's Hospital, ITALY

## Abstract

Carotid intima-media thickness (cIMT) is a surrogate marker of early atherosclerotic changes in children. cIMT-studies are hard to compare, due to variations in ultrasound protocols, especially regarding the common carotid artery (CCA) segment measured in relation to the bulb. This study’s purpose was therefore to compare two distinct CCA segments in children, to see if cIMT values differ substantially according to the site of measurement. cIMT was assessed after power calculation in 30 children (15 girls) aged 8–17, using B-Mode ultrasound (5–13 MHz) at two CCA locations. The first measurement was performed over a distance of 1 cm immediately after the bulb (A), the second 1cm proximal the bulb (B) over the same distance of 1cm length. Means of end-diastolic far wall cIMT were compared between measurement A and B. cIMT in 30 participants was 0.51±0.06 mm for measurement A and 0.51±0.05 mm for measurement B. Results did not differ significantly (p = .947) over a distance of 2 cm after the bulb. According to our results, studies measuring CCA IMT within the first 2 cm, either close to the bulb or further proximal, can be compared. This will improve interpretation of data and application of reference values.

## Introduction

Carotid intima-media thickness (cIMT) is applied as prognostic subclinical marker of atherosclerosis in healthy children and adolescents [1–4] as well as in children in chronic conditions [5–9]. However, cIMT study results are hard to compare due to variations in ultrasound measurement protocols. cIMT measurements can be made at the common carotid artery (CCA), bifurcation (BIF) or internal carotid artery (ICA)–but even if the same arterial segment is referred to, these segments are not identical. Out of 26 studies there is none that applied a uniform scanning protocol (Table 1).

**Table 1:** Summary of ultrasound B-mode studies measuring intima-media thickness in children and adolescents with different protocols.

**Author**	Subjects (females)	Age (years)	IMT (mm±SD) /range (mm)	CCA	ICA	Bulb	Measurement location	Cardiac Cycle	left side	right side	far wall	near wall	Angle	IMT detection	IMT calculation
Aggoun [5]	n = 57	11.1±3	0.5–0.53±0.03	x			1-2cm proximal the BIF	end-diastolic			x		N/A	automatic	mean
Dawson [10]	n = 635 (322)	11–34	0.49±0.04	x	x	x	N/A	N/A	x	x	x	x	3 diff. angles.	N/A	Mean
Iannuzzi [8]	n = 100 (39)	6–14	0.51–0.54	x			1cm distance from BIF	N/A	x	x	x	x	N/A	N/A	max
Ishizu [11]	n = 60 (33)	5–14	0.44±0.05	x			1-2cm proximal the bulb	end-diastolic	x	x	x		ant./ lat.	N/A	mean and max
Jarvisalo [12]	n = 75 (27)	7–14	0.42–0.47±0.03	x		x	1-2cm proximal the bulb, left bulb	end-diastolic	x	x	x		ant. oblique/ lat.	caliper	mean and max
Jarvisalo [6]	n = 88 (33)	11±2	0.42–0.47±0.04	x			1-2cm proximal the bulb	end-diastolic	x	x	x		ant. oblique/ lat.	caliper	mean
Krantz [13]	n = 229 (131)	12–25	0.54–0.56±0.06	x			distal CCA	N/A		x	x		N/A	automatic	N/A
Krebs [14]	n = 100 (53)	5–18	0.54	x			1cm proximal the bulb	N/A	x	x			lat./ post.oblique	automatic	mean and max
Lande [15]	n = 56	10–18	0.53–0.93	x			1cm proximal BIF	N/A	x		x		N/A	N/A	mean
Lavrencic [16]	n = 56 (32)	11–27	0.49–0.71	x			1cm proximal ICA, BIF, 1cm distal BIF	N/A	x	x	x		N/A	N/A	max
Litwin [17]	n = 110 (40)	6–20	0.41–0.45±0.05	x			1-2cm below BIF	N/A	x	x	x		N/A	N/A	N/A
Menees [18]	n = 49 (22)	6–19	0.46±0.04	x			2cm proximal BIF	CRS/ non-CRS	x	x	x		N/A	semi-automatic	mean
Meyer [19]	n = 52 (29)	9–16	0.39±0.05/ 0.49±0.08	x		x	1cm distal the bulb, BIF	N/A	x	x	x	x	N/A	N/A	mean and max
Mittelman [20]	n = 599 (307)	5–20	0.38±0.04	x			1cm proximal BIF	N/A	x		x		N/A	automatic	N/A
Sass [2]	n = 193 (108)	10–24	0.48–0. 5±0.05	x			3cm proximal BIF	N/A	x	x	x		N/A	automatic	mean
Böhm [3]	n = 267 (143)	6–17	0.51	x			8-18mm proximal BIF	end-systolic		x	x		N/A	semi-automatic	mean
Jourdan [1]	n = 247 (127)	10–20	0.38–0.4±0.04	x			1-2cm proximal BIF	N/A	x	x	x		N/A	caliper	N/A
Doyon [4]	n = 1051	6–18	0.37–0.41	x			1-2cm proximal BIF	N/A	x	x	x		N/A	caliper/semi-automatic	N/A
Pauciullo [21]	n = 94 (45)	2–14	0.39–0.4±0.03	x			1cm proximal BIF	systolic	x	x	x	x	ant./post.	N/A	mean and max
Reinehr [22]	n = 124 (75)	9–13	0.4–0.6	x			near BIF	N/A	x	x	x		N/A	N/A	max
Singh [23]	n = 66 (31)	10–18	0.32±0.08/ 0.33±0.05	x			1cm proximal the bulb	N/A	x	x	x		N/A	N/A	N/A
Sorof [7]	n = 32 (7)	13.9	0.64±0.12	x			2cm distal the flow divider	N/A	x	x	x		N/A	digital caliper	max
Tonstad [24]	n = 90 (29)	10–19	0.48±0.07	x			1cm proximal and distal the bulb	end-diastolic		x	x		N/A	N/A	mean and max
Virkola [25]	n = 46 (16)	2.7–19	0.33–0.5±0.1	x			2cm below BIF	end-diastolic		x	x	x	N/A	electronic caliper	N/A
Weberruß [26]	n = 690 (380)	7–17	0.46±0.03	x			1cm proximal the bulb,	end-diastolic	x	x	x		N/A	semi-automatic	mean
Woo [9]	n = 72 (30)	7–12	0.45–0.49±0.04	x			1cm distal the bulb	N/A	x	x	x		ant/ lat./ post.oblique	automatic	max

IMT, intima-media thickness; CCA, common carotid artery; ICA, internal carotid artery; BULB, bulbus; BIF, bifurcation

cIMT was measured either 1-2cm proximal the bifurcation [1, 4, 5, 17], which is slightly different to others, who indicate the same distance (1–2 cm) but proximal to the bulb [6, 11, 12] and to those, measuring only 1 cm proximal to the bifurcation [8, 16, 20, 21] or 1 cm proximal to the bulb [14, 23, 24, 26]. In contrast, Meyer et al. [19] and Woo et al. [9] define their location as the distal 1 cm of the CCA, Sass et al. [2] measured 3 cm proximal the bifurcation. Lorenz et al. (2007) pointed out 6 different CCA segments where cIMT has been measured [27]. Furthermore, there is a significant difference between the left and right carotid artery [28] as well as between measurements made on the near or far wall [29, 30]. cIMT depends on the cardiac cycle with a variation of about 5% between systolic and diastolic phase [31] and differs substantially according to cIMT calculation with the maximum value obtained, mean of maximum or mean value [32]. Just recently, the Association for European Pediatric Cardiology has made recommendations on cIMT measurement in children [33]. So far, comparing different study results is quite impossible, due to lack in rigorous standardized protocols, methodological variation and inhomogeneous study populations [27, 32, 34, 35].

This study investigated if there is a difference in IMT measured at two CCA segments, first, over a distance of 1cm length immediately proximal to the bulb, and second, over a distance of 1cm length proximal to the first segment. The distance over 2cm was chosen according to Engelen et al. (2013), who noted the CCA segment of 0 to 2cm proximal the bulb as most common site of measurement, observed in a study cohort of 24 871 individuals, out of 24 study centers from 13 different countries [35].

To our knowledge, this is the first study comparing cIMT in two different CCA segments in children.

## Patients and Methods

### Study Design and Subjects

This study reexamined a subsample (n = 30) out of the total study population (n = 1017). Data analysis was part of the prevention project “Sternstunden der Gesundheit” that was conducted from October 2012 to July 2013 in the area of Berchtesgadener Land, Germany. The total study population of 1017 healthy children (534 boys/ 483 girls), aged 7–17, was prospectively studied to calculate reference values and to assess cardiovascular risk factors. The study protocol was approved by the ethics commission of the Technische Universität München (5490/12), written informed consent was obtained from parents of all children as well as from children aged ≥ 14 years.

### Sample size calculation

Power calculation (G*Power 3.1.9 for Windows, University Düsseldorf, Germany) with an effect size d = 0.8 and test power of 0.95 revealed a subsample size of 28 subjects which were analyzed twice [36]. To compare a homogenous sample, 30 healthy children were randomly chosen—6 subjects (3 boys/ 3 girls) out of five age groups (8–10/ 10–12/ 12–14/ 14–16/ 16–18).

### cIMT Measurement

cIMT was assessed with B-Mode ultrasound (ProSound Alpha 6, Hitachi Medical Systems GmbH, Wiesbaden, Germany) using a high frequency linear array probe (5–13 MHz) by two investigators. The coefficient of variation (CV) between both examiners was 4.79%, assessed in 27 subjects out of the whole study population (n = 1017), who were examined by both investigators.

After 15 minutes rest patients were examined in supine position, the neck slightly extended and their head turned 45° opposite the site being scanned. cIMT was measured according to the Mannheim consensus [37] on CCA far wall. Of each subject, 4 video loops of at least 3 heart cycles were stored, 2 for the left and 2 for the right CCA. The cardiac cycle was simultaneously controlled with a 3 lead ECG.

### cIMT Analysis

**Fig 1:**
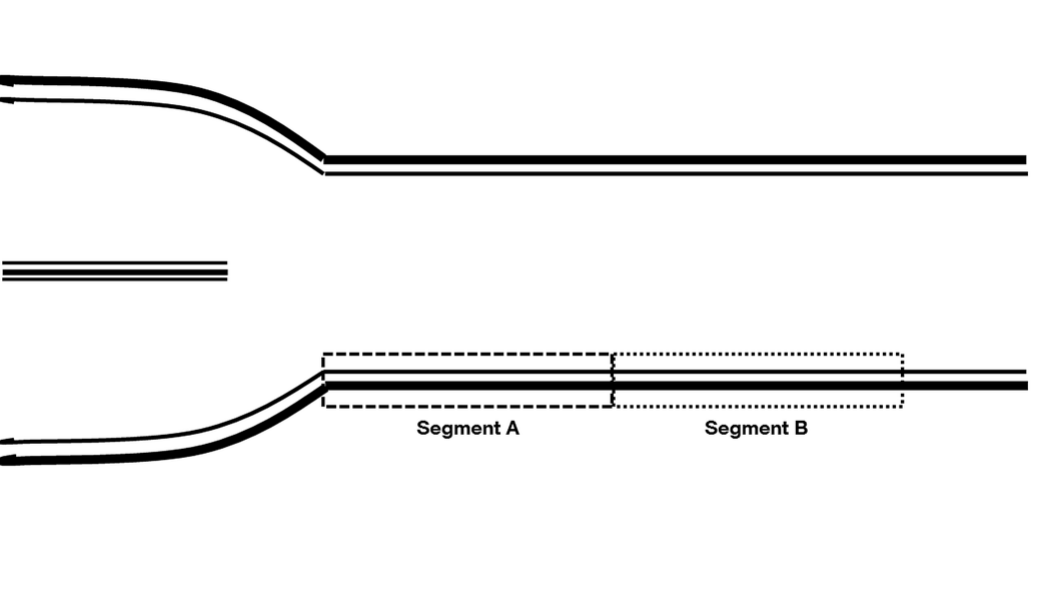
B-Mode Ultrasound image of the common carotid artery. Segment A defines the first measurement about a distance of 1cm immediately proximal to the bulb. Segment B defines the second measurement over the same length of 1cm proximal to segment A.

Out of every video-loop, the best end-diastolic picture was chosen and analyzed twice by the same tester with a semi-automated software (ProSound Alpha 6, Hitachi Medical Systems GmbH, Wiesbaden, Germany) without manual correction. Measurement A refers to the distance over 1cm length immediately proximal to the bulb, measurement B over the same length of 1cm proximal to segment A (Fig 1). For each subject, 4 measurements (2 left and 2 right) were analyzed for segment A and segment B, respectively. cIMT of each measurement was calculated as mean value, the average mean value of analysis A and B, respectively, was calculated and further compared.

### Statistical Analysis

Data was analyzed using IBM SPSS statistics for Windows, version 21.0 (IBM Corp., Armonk, NY, USA). Measurements were compared via intraclass correlation coefficient (ICC), the coefficient of variation (CV), and visualized with a Bland-Altman plot. The CV describes differences between measurement A and B as a percentage of the pooled mean value, calculated according to the following formula:

$$CV=\;\frac{s\;x\;100}{\bar{x}}\%$$

In the formula, *s* is calculated as s=SD/√2 and *SD* is the standard deviation of differences between measurement A and B [38]. As data was normally distributed, Pearson’s correlation coefficient was applied, differences between the two measurements were examined with a t-test for matched pairs. A p-value of < .05 was considered to be statistically significant.

## Results

cIMT was measured in 30 participants (15 boys/ 15 girls). Subject’s mean age was 13±2.98 years. Anthropometric data and cIMT values are shown in Table 2. There were no significant differences between boys and girls in the subsample. Compared to the total study population (n = 1017), the entire subsample as well as boys and girls separately, did not differ significantly regarding BMI and BMI SDS as well as cIMT and cIMT SDS (data not shown). A significant difference in age was observed between the subsample and the total study population (13±3 vs 11.9±2.3, p = .013) and between boys of the subsample and boys of the total study population (13.1±3.1 vs. 11.5±2.1, p = .005).

**Table 2:** Anthropometric data and cIMT values of study participants in mean±SD.

	Boys (n = 15)	Girls (n = 15)	p-value
**AGE [years]**	13.1±3.1	12.9±2.9	.854
**HEIGHT [cm]**	158.4±17.2	152.1±12.2	.260
**WEIGHT [kg]**	53±23.2	46.5±12.1	.653
**BMI [kg/m**^**2**^**]**	19.7±3	20.2±4.9	.935
**BMI SDS**^a^	0.26±1.1	0.23±0.9	.928
**cIMT [mm]**	0.47±0.04	0.47±0.04	.804
**cIMT SDS**	-0.02±1.16	0.21±1.02	.566

^a^ SDS is for Standard Deviation Scores, according to the german reference population for Body Mass Index (BMI) [39] and carotid intima-media thickness (cIMT) [26].

**Fig 2:**
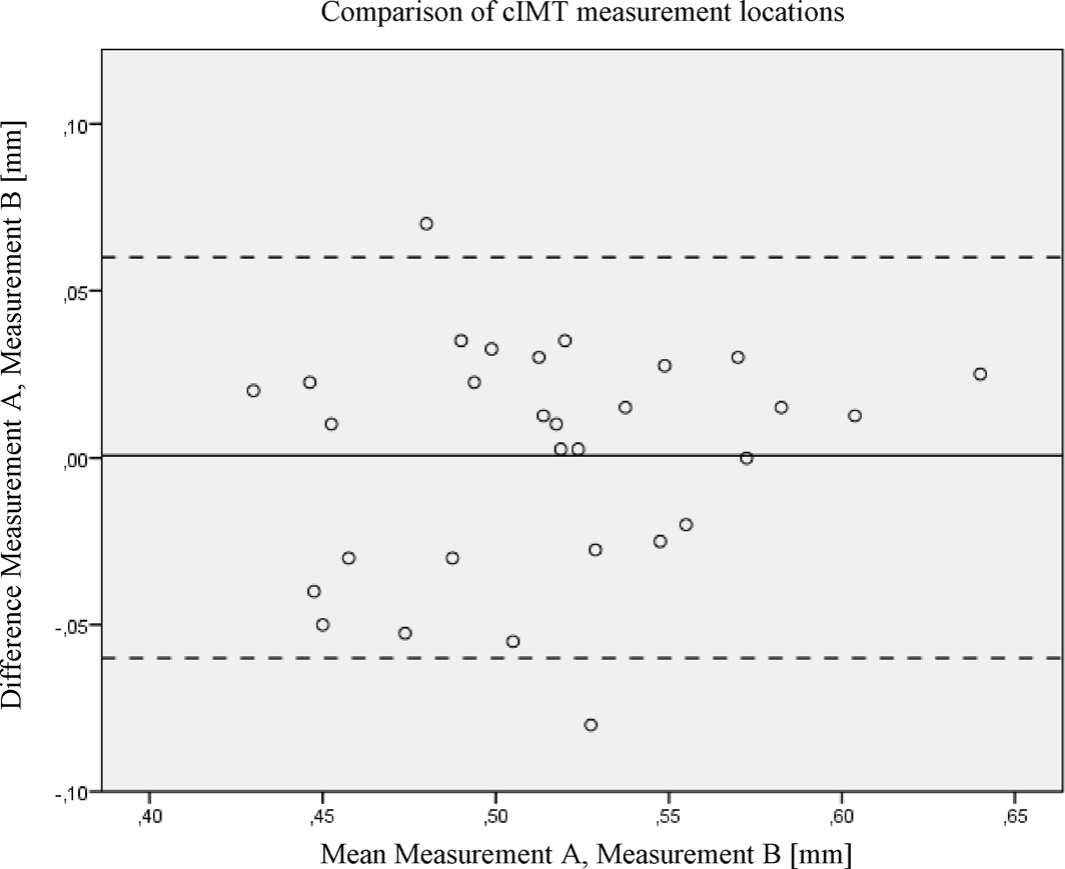
Bland-Altman analysis of measurement A and B.

Mean cIMT for measurement A was 0.51±0.06mm and 0.51±0.05mm for measurement B, respectively. The average measure ICC between measurement A and B was 0.887 with a 95% confidence interval from .764 to .949 (p < .001), CV was 2.6%. The Bland-Altman analysis visualizes the level of agreement between analysis A and B (Fig 2). The x-axis displays the common mean value for measurement A and B in each subject, the y-axis the corresponding difference between measurement A and B. The upper and lower lines represent the 95% limits of agreement, which ranges from -0.069 mm to 0.067 mm.

Pearson’s correlation coefficient between the two measurements was r = 0.795 (p < .001) for the entire sample and r = 0.782 (p = .001) for boys and r = 0.814 (p < .001) for girls.

There were no significant differences between cIMT measured at segment A and B, respectively (p = .947; 95% CI = ±0.01mm) in the t-test for matched pairs within the entire sample neither for boys and girls separately (p = .923; 95% CI = ±0.02mm for boys and p = .761; 95% CI = -0.014/+0.018 for girls, respectively).

## Discussion

Measurement protocols of cIMT values differ very much among studies, which makes comparison of results quite impossible [34]. To enhance comparability of results, this study’s purpose was to analyze cIMT measured in two distinct CCA segments in children. The first segment was chosen over a distance of 1cm length immediately proximal to the bulb, and second, over the same distance of 1cm length proximal to the first segment. Our results revealed no statistical differences in mean cIMT between the two CCA segments, measured in 30 children on the left and right CCA far wall. To our knowledge, this is the first study to compare cIMT in two different CCA segments in children.

Kornet et al. [40] compared two CCA points in an adult cohort, immediately after the bulb and 20–30 mm more proximal. Opposite to our results, they state a larger cIMT closer to the bulb than 20–30 mm more proximal and assume near-wall shear rate (WSR) as possible explanation. In sites of lower WSR, which is true for the area close to the bulb, cIMT is supposed to be larger than in sites of higher WSR.

In our study, cIMT was assessed with B-mode, whereas Kornet et al. [40] applied radio frequency signal analysis in M-mode. This technique only measures a single point of thickness that does not represent the arterial status adequately [41] and might account for controversial results. Progress in ultrasound technology with a far more accurate assessment nowadays could be another reason as well as participants’ age. We assessed cIMT in children and adolescents aged 8–17, whereas participants in the study by Kornet et al. [40] were adults (18–67 years). According to De Groot et al. [42], there is a smaller population variability in thinner arterial walls, which is the case in children. Furthermore, atherosclerotic changes develop slowly over decades, thus structural differences in children’s cIMT might not be as developed as in adults [43].

With the exception of the study by Kornet et al. [40], studies comparing different IMTs did not focus on different segments of the same arterial part but on (a) differences between CCA, ICA and the bulb region [43, 44] or (b) on differences between CA and the femoral artery [2, 45] or abdominal aorta [10] and their association to cardiac risk factors. Meyer et al. (2006) measured cIMT in the CCA and bifurcation area (BIF) in normal weight children, where mean cIMT varied between CCA and BIF from 0.39±0.05mm (CCA) to 0.43±0.07mm (BIF), respectively, but did not report if this difference was significant.

## Conclusion

Our results ensure comparability of studies in children and adolescents with cIMT measurements at different CCA locations over a distance of 0–2 cm proximal to the bulb. CCA cIMT is frequently assessed within this distance, as observed in a study cohort of more than 20 000 individuals [35]. The Association for European Paediatric Cardiology tried to unify cIMT measurements in children [33]. In these guidelines, they recommend to measure cIMT at segment A, which is sometimes difficult to detect in children, as the bulb is located beyond the jawbone. In these cases, it is of special importance to know, that segment A and B (S1 Table) do not differ significantly in children and both measurements represent a reliable cIMT.

## Limitations of the Study

As a limitation of our study we did not measure the pubertal status of our participants. Only one study could be found, addressing pubertal status and cIMT, but in children with type 1 diabetes. In this work, cIMT did not increase during puberty [46]. Results in the literature are inconsistent about a significant influence of age on cIMT [1–4, 11]. Within our sample, we observed no significant age influence on cIMT [26].

## Supporting Information

S1 Table cIMT [mm] at Segment A and B. Segment A refers to the measurement immediately proximal to the bulb and Segment B to 1cm proximal to Segment A.
(XLSX)
